# Effect of Austempering Processes on the Tensile Properties and the Work-Hardening Behavior of Austempered Bainitic Steels Below the Martensite Start Temperature

**DOI:** 10.3390/ma16165562

**Published:** 2023-08-10

**Authors:** Kun Wang, Feng Hu, Wen Zhou, Serhii Yershov, Li Li, Kaiming Wu

**Affiliations:** 1 The State Key Laboratory of Refractories and Metallurgy, Collaborative Innovation Center for Advanced Steels, International Research Institute for Steel Technology, Wuhan University of Science and Technology, Wuhan 430081, China; wangkun120306@163.com (K.W.);; 2 Zhejiang Tsingshan Iron & Steel Co., Ltd., Qingtian 323903, China; 3 Metals Valley & Band (Foshan) Metallic Composite Co., Ltd., Foshan 528000, China

**Keywords:** austempered bainitic steel, tensile property, ductility, work-hardening behavior

## Abstract

The tensile properties and work-hardening behavior of austempered bainitic steels below martensite start temperature (Ms) were investigated and compared with those of bainitic steel austempered above Ms. The results show that the tensile strength and yield strength increased from 1096 MPa and 734 MPa to 1203 MPa and 951 MPa, respectively, when the austempering temperature was decreased from 400 °C to 300 °C. However, the total elongation decreased from 23% to 16%. The martensite-retained austenite blocks and bainitic ferrite laths are significantly refined. With a decrease in the austempering temperature, the volume fraction of retained austenite decreased from 15.4 vol% to 6.2 vol%. The carbon content in retained austenite increased from 1.12 wt% to 1.69 wt%. All tensile specimens exhibited three stages of deformation in the differential Crussard−Jaoul (C−J) models. The difference in ductility is mainly attributed to the transformation of the retained austenite blocks into strain-induced martensite during deformation. The initial content of retained austenite is the main factor affecting the ductility of bainitic steels. Therefore, the work-hardening ability of austempered bainitic steel above Ms is higher than that of bainitic steel below Ms.

## 1. Introduction

Recently, micro-nanostructured bainitic steels composed of bainitic ferrite (BF) laths and retained austenite (RA) films have attracted extensive attention due to their excellent strength-ductility balance [1,2,3,4,5,6]. The mechanical properties are associated with the regulation of the multiphase microstructures in bainitic steels. The optimal combination of strength, ductility, and toughness was obtained by combining austempering and partitioning processes by Gao et al. [7]. The excellent mechanical properties are attributed to the refined film-like RA in the steel. Zhou et al. [8] reported the inhibitory effect of the morphology and stability of RA on crack initiation and propagation during tensile deformation.

Since Radcliffe and Rollason et al. [9] confirmed the isothermal transformation product below Ms was bainite, the phase transformation and mechanical properties of bainitic steel below Ms have become the focus of research, and the transformation kinetics and crystallographic characteristics have been extensively studied [10,11,12]. Xia et al. [13] obtained a multiphase microstructure composed of martensite, bainite, and retained austenite by different austempering temperatures below Ms, and achieved the optimal mechanical properties of a tensile strength of 1512 MPa, a total elongation of 15.4%, and impact toughness of 119 J/cm^2^. The enhanced ductility and toughness are attributed to regulating the volume fractions of martensite, bainite, and retained austenite.

In our previous study [14], the BF laths and martensite-retained austenite (M-RA) blocks were refined by austempering below Ms in 0.3C steel, and both the strength and toughness of bainitic steel were improved. Zhao et al. [11] reported that a refined bainite structure in low-carbon steel was obtained by austempering below Ms. Moreover, in their other work [15], a low-carbon ultra-fine bainitic steel with an ultimate tensile strength of 1715 MPa and an impact toughness of 180 J/cm^2^ was obtained by austempering below Ms. According to the existing research results, the austempering process below Ms is an effective heat treatment process that can obtain an ideal balance of the high strength and toughness. Nevertheless, the effect of the austempering process below Ms on the ductility of bainitic steels remains to be studied in detail. Because in the existing research, satisfactory improvement in ductility has yet to be obtained.

García-Mateo and Caballero [16] proposed that the ductility of high-carbon bainite steels is dominated by RA, and the chemical stability of RA significantly influences ductility. In addition, García-Mateo et al. [17] also improved the stability of RA by adding Si elements, thus obtaining a satisfactory combination of strength and ductility with an ultimate tensile strength of 2 GPa and a total elongation higher than 20%. Misra et al. [18] reported that the carbon distribution during the isothermal transformation directly affects the RA stability. The carbon distribution is reflected in the bainite isothermal transformation process and is similar to the carbon partition mechanism of the Q&P process [13,14,19,20]. In Q&P steels, carbon-rich RA obtained using carbon partitioning also plays a vital role in the work-hardening process [19]. Chhajed et al. [21] improved the RA stability by controlling the prior austenite grain (PAG) size, and the toughness and ductility of nanostructured bainitic steels were improved by increasing the PAG size. Thus, in bainitic steels, it can be determined that ductility is strongly associated with the stability and distribution of RA, as this involves transformation-induced plasticity (TRIP) effects during deformation.

The purpose of this paper is to investigate the effect of austempering processes above/below Ms on the tensile properties of bainitic steels and to analyze the effect of retained austenite on the ductility of different austempered bainitic steels. Finally, the multistage work-hardening behavior of the tensile stress–strain curve is elucidated by several empirical equations. This work will provide theoretical guidance for the application of high-performance steel materials in the field of industrial production.

## 2. Materials and Methods

**Figure 1:**
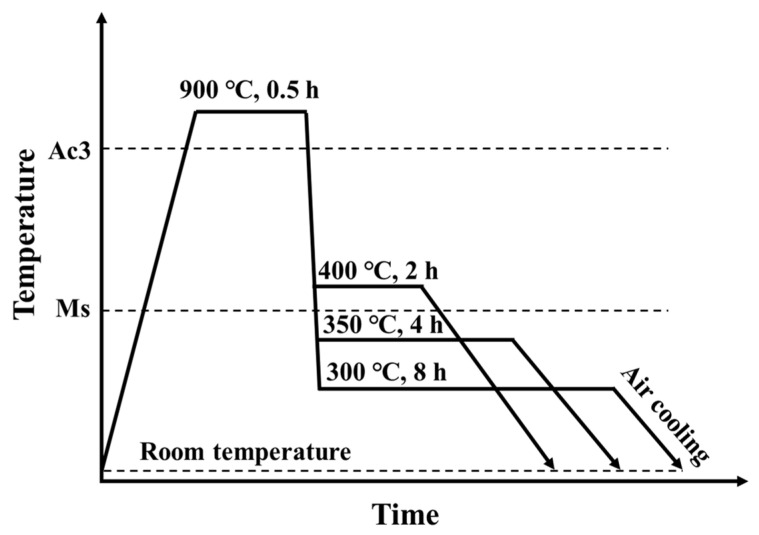
The schematic diagram of the heat treatment process.

The chemical composition of the investigated steel is listed in Table 1. The martensite start temperature (Ms = 360 °C) of the investigated steel was measured in a previous study [14]. Metallographic and tensile specimens were cut from billets using a wire-cutting machine. The schematic diagram of the heat treatment process of the studied steel is shown in Figure 1. First, the homogenized annealed samples were austenitized at 900 °C for 0.5 h. Subsequently, they were rapidly cooled to different austempering temperatures for bainite transformation (400 °C for 2 h, 350 °C for 4 h, and 300 °C for 8 h). Finally, all steel samples were air-cooled to room temperature. According to the austempering temperature, the heat-treated samples were marked as AT400, AT350, and AT300, respectively.

Details of the tensile testing and microstructural characterization methods are detailed in previous studies [14,22]. This paper will not repeat them.

## 3. Results and Discussions

### 3.1. Mechanical Properties

**Figure 2:**
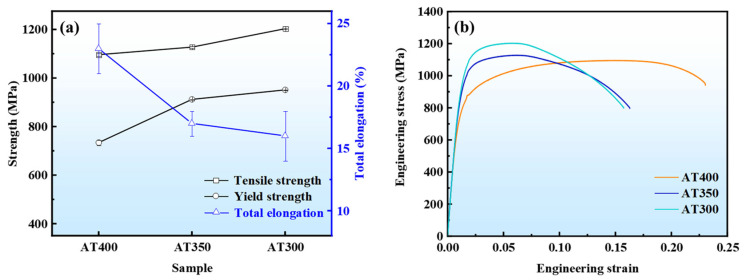
(**a**) Tensile properties of different experimental steels, (**b**) engineering stress-engineering strain curves.

Figure 2 presents the tensile properties and the engineering stress-engineering strain curves of the experimental steels. The tensile strength and yield strength of AT400 are 1096 MPa and 734 MPa, respectively. As the austempering temperature decreases, the tensile strength and yield strength of the experimental steels gradually increase. The tensile strength and yield strength of AT350 are 1127 MPa and 912 MPa, respectively. The tensile strength (1203 MPa) and yield strength (951 MPa) of AT300 are higher than those of AT400 and AT350. The increase in strength is attributed to the increase in the content of the hard phase and the decrease in the content of the soft phase in the experimental steels. As the austempering temperature decreases, the driving force for the transformation from γ phase to α phase increases. Therefore, the volume fraction of bainite increases while the volume fraction of retained austenite decreases. In addition, the formation of α-phase increases the dislocation density. The mutual entanglement of dislocations increases the resistance to dislocation movement, resulting in an increase in strength as the austempering temperature decreases. The AT400 sample had the highest total elongation (23%). The total elongation of the experimental steel decreases with the decrease in the austempering temperature. The total elongation of AT350 and AT300 was 17% and 16%, respectively. The influence of the microstructure of the experimental steels on the elongation and work-hardening behavior will be analyzed in detail below.

### 3.2. Microstructure

**Figure 3:**
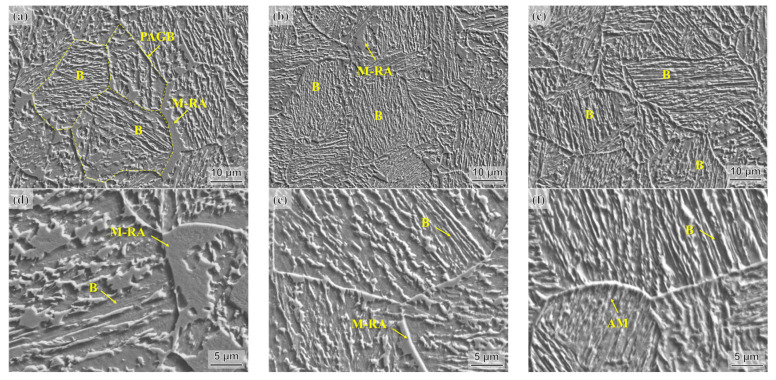
SEM micrographs of experimental steels before tensile test: (**a**,**d**) AT400, (**b**,**e**) AT350, (**c**,**f**) AT300.

Figure 3 presents representative microstructures of three heat-treated steel samples before the tensile test. In the SEM micrograph of the AT400 sample (Figure 3a,d), in addition to the lamellar bainite, large-sized M-RA blocks distributed at the prior austenite grain boundaries (PAGBs) and within the prior austenite grains (PAGs) are also observed. As the austempering temperature decreases, the M-RA blocks and the lamellar bainite in the AT350 sample are refined (Figure 3b,e). Only small-sized M-RA blocks distributed at PAGBs are observed in the AT350 sample. In the AT300 sample, few obvious M-RA blocks are observed (Figure 3c,f).

**Figure 4:**
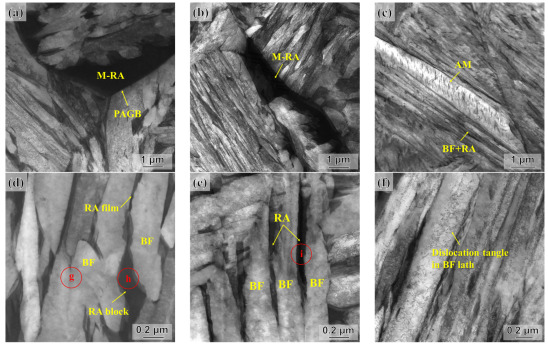

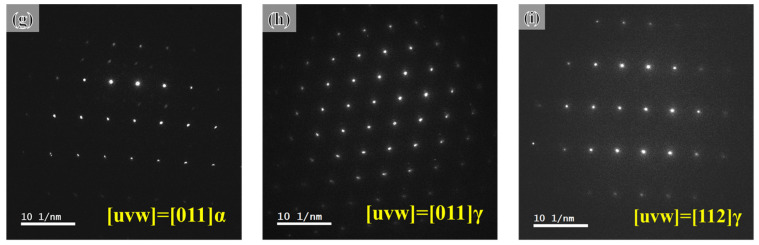
TEM micrographs of experimental steels. (**a**,**d**) AT400, (**b**,**e**) AT350, (**c**,**f**) AT300. (**g**,**h**,**i**) are the selected area electron diffraction patterns of γ phase and α phase in (**d**,**e**), respectively. Note: The red circles represent the selected areas of the electron diffraction patterns.

Figure 4 presents the TEM micrographs of different experimental steels before the test. Irregular M-RA blocks are distributed at PAGBs in AT400 and AT350 samples (Figure 4a,b). In the AT300 sample, these M-RA blocks almost disappear. Only BF laths and RA films are observed near the athermal martensite (AM) laths (Figure 4c). The selected area electron diffraction patterns confirm the BF (white) and RA (black) in Figure 4d,e. Long isothermal times cause carbide precipitation in AM laths, and thus, BF and AM can be easily distinguished. Figure 4f shows the dislocations in the BF laths. Dislocation slip plays an important role in ductility during deformation. As the austempering temperature decreases, the M-RA blocks and the microstructures composed of BF laths and RA films in AT350 and AT300 samples are refined. In our previous study [14], quantitative statistics of M-RA islands were performed by SEM micrographs. The average equivalent diameters of the M-RA islands in AT400, AT350, and AT300 samples were 3331 ± 258 nm, 2688 ± 210 nm, and 1856 ± 180 nm, respectively. The average widths of BF laths were quantified by TEM micrographs. The mean diameters of BF laths in AT400, AT350, and AT300 samples were 362 ± 78 nm, 312 ± 56 nm, and 281 ± 49 nm, respectively.

Coarse bainite laths and M-RA blocks are obtained in the austempered bainitic steel AT400, which is related to the high austempering temperature. When the austempering temperature is reduced below the Ms temperature, the M-RA blocks and BF laths are refined. The M-RA blocks almost disappear in the AT300 sample, and a refined microstructure is obtained. This is attributed to the increase in the driving force for the phase transformation. The driving force of phase transformation refers to the decrease in the Gibbs free energy (Δ*G*) of the system caused by the generation of the new phase (α) from the parent phase (γ phase) under the condition of constant pressure and temperature. The relationship between Δ*G* and the supercooling is shown in Equation (1).

(1) $$\Delta G=L\left(T_{m}-T\right)/T$$

where *L* is the latent heat of phase transformation per unit volume, *T_m_* is the equilibrium temperature of the two phases, *T* is the thermodynamic temperature at which the phase transformation occurs, and the supercooling Δ*T* = *T_m_* − *T*. *L* and *T_m_* are constants related to materials. In this paper, when the austempering temperature *T* decreases, the supercooling Δ*T* increases, and the Gibbs free energy Δ*G* increases. Therefore, the phase transformation driving force increases as the austempering temperature decreases. The high supercooling provides a sufficient driving force for bainite phase transformation [15]. In addition, when cooling from the austenitizing temperature to the austempering temperature, the athermal martensite formed first increases the dislocation density and the α/γ interface in the PAGs, which provides potential nucleation sites for the subsequent bainite transformation [10,12]. Therefore, the austempering process below Ms can promote the bainite transformation and refine the microstructure.

### 3.3. XRD Analysis

**Figure 5:**
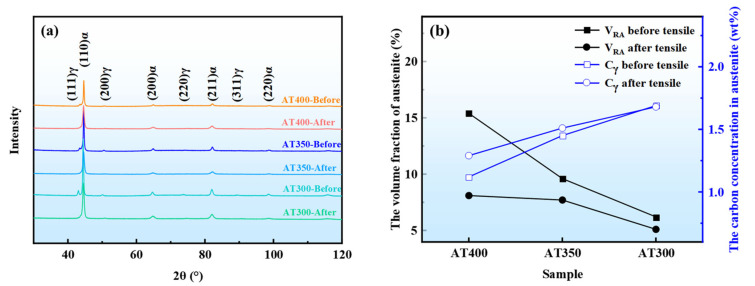
(**a**) XRD profiles of experimental steels before/after the tensile test, (**b**) the RA volume fraction and the carbon concentration in RA before/after the tensile test. Note: V_RA_ is the volume fraction of retained austenite, and C_γ_ is the carbon content in retained austenite.

Figure 5a shows the XRD profiles of tensile specimens before and after the tensile test. The RA volume fraction and the carbon concentration in RA are quantitatively analyzed by XRD (Figure 5b). Before the tensile test, the RA volume fraction in the AT400 sample (15.4 vol%) was higher than that in the AT350 (9.6 vol%) and AT300 samples (6.2 vol%) due to the high austempering temperature [11,12,13]. The lower the austempering temperature, the greater the driving force of the phase transformation. Therefore, as the austempering temperature decreases, the RA volume fraction decreases. The carbon contents of RA in AT400, AT350, and AT300 samples were 1.12 wt%, 1.45 wt%, and 1.69 wt%, respectively. As the austempering temperatures decreases, the RA blocks were refined. The low-stability RA blocks were reduced, and the high-stability RA films were preserved [11,13]. At low austempering temperatures, the driving force of phase transformation increases. Furthermore, more carbon diffuses from the α phase into the γ phase during the isothermal process. Therefore, the carbon content of the interlath RA films increases. In addition, Al and Si elements were added to the investigated alloys to suppress carbide precipitation, which further promotes the enrichment of carbon in RA. Therefore, as the austempering temperature decreases, the RA volume fraction in the AT350 and AT300 samples decreases, and the carbon content in RA increases.

After the tensile test, the RA volume fraction in the AT400 sample was reduced to 8.1 vol%. This indicates that about 47.4% of the RA was transformed into martensite during deformation. The RA transformations in the AT350 and AT300 samples were 19.8% and 17.7%, respectively. This reduction may be related to the high-stability RA, which requires greater strain accumulation during deformation to trigger the TRIP effect. After the tensile test, the carbon content of the RA in the AT400 and AT350 samples was slightly increased. The low-stability RA tends to undergo martensitic transformation first during deformation, while the high-stability RA films require greater strain accumulation to begin to undergo phase transformation. Therefore, the RA with higher carbon content is more likely to be retained after the tensile test, which leads to a slight increase in the average carbon content in RA.

### 3.4. Tensile Fracture Morphology

**Figure 6:**
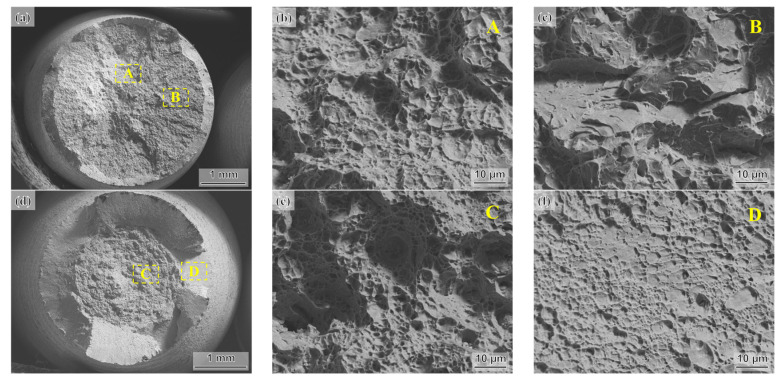

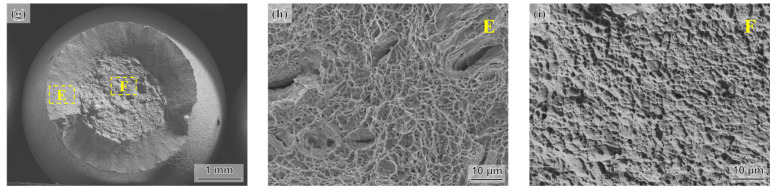
SEM micrographs of fracture surfaces for (**a**–**c**) AT400, (**d**–**f**) AT350, and (**g**–**i**) AT300. Note: The same capital letters represent the corresponding observation area.

Figure 6 presents the SEM morphologies of the fracture surfaces of different tensile specimens. In the AT400 sample, the fracture features in the central area of the fracture surface include cleavage planes and small dimples (Figure 6b). In the dotted box B, the fracture features are mainly river patterns and secondary cracks (Figure 6c). This indicates that the fracture mode near the dotted box B is a brittle fracture. Therefore, the fracture mechanism of the AT400 sample is a mixed fracture mode combining ductile fracture and brittle fracture. The fracture morphology of the AT350 sample is a cup–cone fracture (Figure 6d). An obvious shear lip area was observed on the fracture surface. Dimples of various sizes were observed in dashed box C (Figure 6e). In the dotted box D, the fracture is characterized by shallow and dense dimples (Figure 6f). In the central area of the AT350 sample, the fracture morphology is dense dimples of different depths (Figure 6e). The shear lip area is distributed with shallow dimples (Figure 6f). This indicates that the fracture mode of the AT350 tensile specimen is a typical ductile fracture. A similar cup–cone fracture was also observed in the AT300 sample (Figure 6g). Densely distributed dimples and a small number of secondary cracks were observed in the central area of the fracture surface (Figure 6h). The fracture features of the shear lip area of AT300 are fine and dense shallow dimples (Figure 6i). Therefore, the fracture modes of the AT350 and AT300 samples are both ductile fractures. However, the AT350 and AT300 samples did not show high total elongation in the tensile test. In contrast, the highest total elongation was obtained in the AT400 sample.

**Figure 7:**
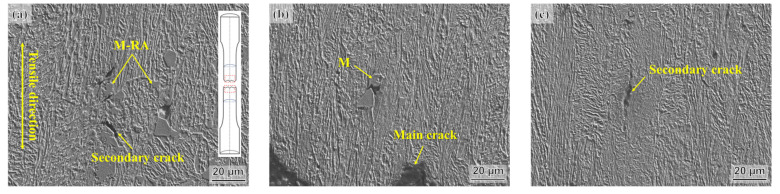

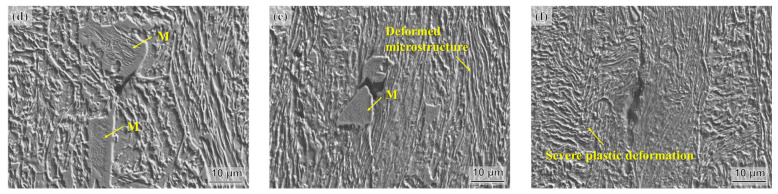
SEM micrographs of longitudinal section of tensile specimens after tensile test: (**a**,**d**) AT400, (**b**,**e**) AT350, (**c**,**f**) AT300. Note: The red rectangle in (**a**) is the SEM observation areas of the tensile specimens.

The cleavage fracture in the AT400 sample may be attributed to the TRIP effect of the low-stability RA into fresh martensite during deformation, which relieves the local stress concentration. However, the brittle martensite blocks also provide potential nucleation sites for crack initiation. Additionally, the fresh martensite content in the AT400 sample is higher than that in the AT350 and AT300 samples after tensile tests. This means that the cracks in the AT400 sample are more likely to nucleate inside or at the edge of the fresh martensite blocks, as shown in Figure 7b,d. Therefore, the cleavage fracture features in AT400 may be associated with brittle martensite blocks. Nevertheless, the TRIP effect still provides excellent ductility during deformation in the AT400 sample. This suggests that the cracking of brittle martensite may mainly occur at the end of the tensile test, while before that, most of the strain is absorbed via the TRIP effect.

Figure 7 presents the SEM micrographs of the tensile fracture section after the tensile test. Due to the uniaxial tension, the microstructures of all tensile specimens were elongated along the tensile direction. The microstructures of these samples underwent obvious plastic deformation. In the AT400 and AT350 samples, secondary cracks distributed near the M-RA block were observed. Cracking mainly occurs inside or at the edge of the M-RA blocks (Figure 7d,e). The low-stability RA blocks are easily transformed into martensite via the TRIP effect during deformation. Because the coordinated deformation ability between the soft phase and the hard phase is low, the interfaces between the hard martensite and the soft ferrite/austenite may debond under the action of tensile stress, thereby causing crack initiation and expansion. In addition, cracks penetrating martensitic blocks were found near the fracture (Figure 7e). These cracks tend to initiate inside the brittle martensite islands. Ghadbeigi et al. [23] pointed out that during the stretching process, cracks caused by local stress concentration initiate and propagate in the martensite islands and may cause the fracture of the martensite islands. Since few obvious M-RA blocks are inside the AT300 sample, cracks may tend to initiate near inclusions or voids and propagate along the tensile direction (Figure 7c,f).

### 3.5. Theoretical Model of the Work-Hardening Behavior

In order to further analyze the tensile behavior of austempered bainitic steel, the Hollomon, differential C−J, and modified C−J flow stress models used to describe various alloy materials were selected to analyze the work-hardening behavior of the tensile stress–strain curve. Hollomon analysis model is widely used in the analysis of the work-hardening rate of alloy materials [24,25,26]. Hollomon analysis describes the stress–strain curve as

(2) $$\sigma =k\varepsilon^{n}$$

where *σ* is the true stress, *ε* is the true strain, *k*, and *n* values are determined by the slope and intercept of an ln *σ* − ln *ε* plot. The ln arithmic form of Equation (3) is as follows.

(3) ln⁡σ=nln⁡ε+ln⁡k

Crussard and Jaoul [27,28,29] proposed a differential C−J analysis based on the Ludwik relationship. The Ludwik formula related to the true stress-true strain curve is as follows.

(4) $$\sigma =\sigma_{0}+k_{1}\varepsilon^{n_{1}}$$

where *σ* is the true stress, *ε* is the true strain, *n*_1_ is the work-hardening exponent, *σ*_0_ and *k_1_* are constants related to the material. The ln arithmic form of Equation (5), differentiated with respect to *ε*, is as follows.

(5) $$\ln\left(d\sigma /d\varepsilon \right)=\ln\left(k_{1}n\right)+\left(n_{1}-1\right)\ln\varepsilon$$

The modified C−J analysis method based on the Swift equation has been widely used in analyzing the work-hardening behavior of single-phase and dual-phase steels [25,26,30,31,32]. The modified C−J analysis can reflect the work-hardening behavior in different stages accurately. The Swift formula for describing stress–strain relationship is as follows [33].

(6) $$\varepsilon =\varepsilon_{0}+p\sigma^{q}$$

where *ε*_0_ and *p* are materials constants, *q* is the inverse of the work-hardening exponent. The ln arithmic form of Equation (7), differentiated with respect to *ε*, is as follows.

(7) $$\ln\left(d\sigma /d\varepsilon \right)=\left(1-q\right)\ln\sigma -\ln\left(pq\right)$$

where (1 − *q*) is the slope of a ln (*dσ*/*dε*) − ln *σ* plot, −ln (*pq*) is the intercept of a ln (*dσ*/*dε*) − ln *σ* plot.

**Figure 8:**
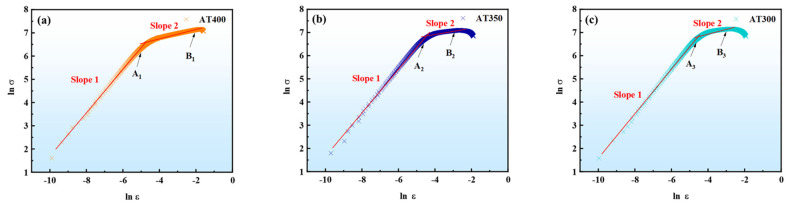

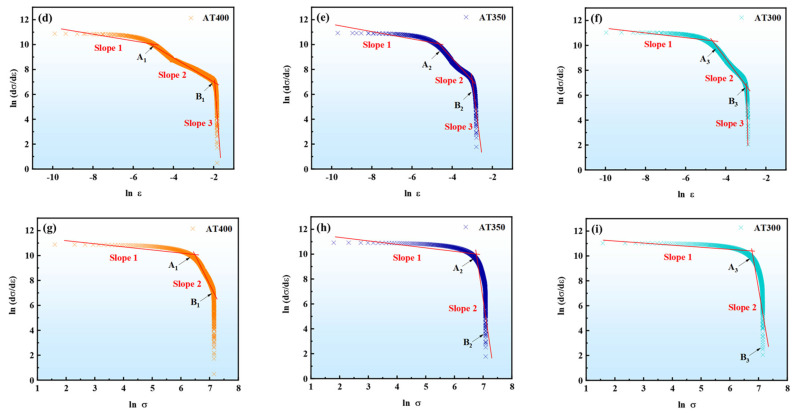
Hollomon analysis of the true stress-true strain curves for (**a**) AT400, (**b**) AT350, and (**c**) AT300. Differential C−J analysis of the true stress-true strain curves for (**d**) AT400, (**e**) AT350, and (**f**) AT300. Modified C−J analysis of the true stress-true strain curves for (**g**) AT400, (**h**) AT350, and (**i**) AT300. Note: Point A_i_: the ends of the linear elastic stage. Point B_i_: ultimate tensile strength point.

Figure 8a–c present the results of the Hollomon analysis of three tensile specimens. Although Hollomon analysis is one of the commonly used empirical formulas for stress–strain curves, all the ln *σ*-ln *ε* plots in Figure 8 do not conform to the linear Hollomon empirical formula (Equation (3)). The multistage slope values of the Hollomon analysis curves of different tensile specimens are listed in Table 2. The ln *σ*-ln *ε* plots follow a linear relationship in the linear elastic phase. However, when ln *ε* increases, all the curves gradually deviate from linearity. Therefore, a single value of *n* cannot be assigned to the entire ln *σ*-ln *ε* curve in this study. The slope of the linear elastic phase and the work-hardening phase need to be discussed in stages. Therefore, a multistage analysis of the work-hardening behavior during deformation was carried out by differential C−J and modified C−J models, respectively.

Figure 8d–f present the ln (d*σ*/d*ε*) − ln *ε* plots for three tensile specimens. The nonlinear changes of the differential C−J plots show that, with an increase in strain, the work-hardening behavior presents three work-hardening stages. The (n_1_ − 1) value decreases with increasing strain. The value range of the first stage (n_1_ − 1) is −0.19~−0.24. Deformation at this stage is associated with dislocation slip and dislocation propagation. The value range of the second stage (n_1_ − 1) is −0.91~−4.05. At this stage, the slope of the AT400 sample (−0.91) was higher than that of the AT350 (−4.00) and AT300 samples (−4.05). Moreover, the second phase of the AT400 sample exhibited a longer variation curve with increasing strain. This indicates that at this stage, the work-hardening rate of the AT400 sample is higher than that of the AT350 and AT300. Compared with the AT350 and AT300, the AT400 has sufficient work-hardening behavior at this stage. Although there is a slight kink in the curve of the second stage, it can be considered that this stage has the same work-hardening exponent. This bend corresponds to the yield plateau in the engineering stress-engineering strain curve.

Studies have shown that negative values of *n* may be related to high-stress fields inside the material due to increased dislocation density and/or microstructural inhomogeneity [31,34]. After the ultimate tensile strength appears, the value of n decreases rapidly. This means a rapid loss of work-hardening ability. At this stage, there is a cooperative deformation among bainitic ferrite, martensite, and retained austenite [30,31]. However, the stress concentration and the rapid increase in the lattice defect density will inevitably lead to the fracture of the tensile specimen. Therefore, the value of (n_1_ − 1) in the third stage decreases rapidly with increasing strain. This corresponds to the end of the work-hardening behavior during the tensile test. Although the (n_1_ − 1) value of the AT400 sample is greater than that of the AT350 and AT300 samples, since the work-hardening rates of the three samples are very low at this stage, the difference in differential C−J plots is mainly reflected in the second stage. The third stage is related to the cooperative deformation of α and γ phases before fracture. Severe plastic deformation and accumulation of local stress eventually caused the fracture of the tensile specimen.

Figure 8g–i present the ln (d*σ*/d*ε*) − ln *σ* plots for three tensile specimens. Three curves were analyzed using the modified C−J analysis method. The modified C−J analysis shows that the deformation of the tensile specimen proceeds in two stages. The slopes (Slope 1) of the three curves in the linear elastic stage are very close (−0.20~−0.25). The difference in plots is mainly reflected in the second stage (Slope 2). At this stage, the slope of AT400 (−3.99) was greater than that of the AT350 (−19.42) and AT300 samples (−18.14). This indicates that the work-hardening rate of the AT400 is higher than that of the AT350 and AT300 at this stage. The work-hardening rates of AT350 and AT300 samples are close at this stage. The curve after the ultimate tensile strength point (B_i_) is almost perpendicular to the ln σ axis. Therefore, the tensile specimen fractures very quickly when the peak stress is reached. Almost no significant work-hardening occurs.

**Figure 9:**
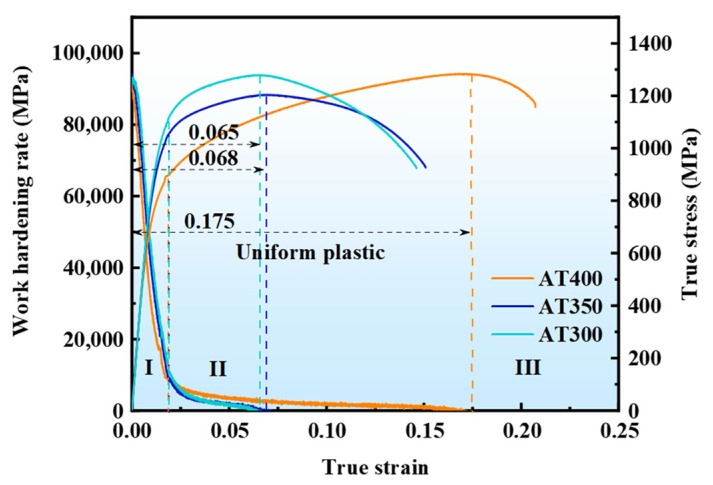
True stress-true strain curves and work-hardening rate curves of different experimental steels. Note: Roman numerals represent different stages during the tensile tests, respectively.

Figure 9 presents the true stress-true strain curves and work-hardening rate curves for different tensile specimens. The results show no significant difference in the work-hardening rates of the three tensile specimens at the beginning of the tensile process. When the true strain increases gradually, the work-hardening rate of the AT400 specimen is higher than that of the AT350 and AT300 specimens, and its work-hardening behavior continues to the peak stress. The true stress-true strain curves show that the AT400 sample has a longer uniform plastic deformation stage compared with the AT350 and AT300 samples. As the strain increases, plastic deformation of retained austenite occurs first. The differences in the work-hardening rates of the three specimens are mainly reflected in the second stage. In the true stress-true strain curve, the second stage begins at the end of the elastic deformation stage and ends after the peak stress occurs. The early plasticity comes from the plastic deformation of the γ phase and the elastic deformation of the α phase. As the strain increases, the dislocation density in the retained austenite increases. These dislocations serve as potential nucleation sites for fresh martensite during subsequent stretching. As the strain increases, the low-stability retained austenite transforms into fresh martensite [35,36]. The carbon content in the retained austenite of the AT400 is lower than that in the AT350 and AT300 samples. Moreover, the volume fraction of blocky RA in the AT400 sample is higher. However, the austempering process below Ms refined the microstructures of AT350 and AT300 samples and reduced the volume fraction of blocky RA. BF laths and RA films are also refined. Shen et al. [37] reported that the initial RA content was closely related to the quantity of RA to M transformation. Therefore, in the early stage of deformation, the transformation quantity of RA in AT400 samples is higher than that in AT350 and AT300, and the TRIP effect in AT400 samples occurs earlier than in AT350 and AT300 samples. In addition, with the accumulation of strain, some high-stability retained austenite films may undergo martensitic transformation in the middle and late stages of deformation. The transformation of retained austenite to martensite can relieve local stress concentration, thereby improving the plasticity of the material. Therefore, compared with the AT350 and AT300 samples, the AT400 sample has a higher work-hardening rate and a longer work-hardening plateau under high strain.

## 4. Conclusions

(1) As the austempering temperature decreases, the tensile strength and yield strength of the experimental steels increase, and the total elongation decreases. The highest tensile strength (1203 MPa) and yield strength (951 MPa) were obtained in the AT300 sample. The highest total elongation (23%) was obtained in the AT400 sample.

(2) The microstructures of experimental steels are composed of bainite, martensite, and retained austenite. BF laths and M-RA blocks are significantly refined by austempering below Ms. As the austempering temperature decreases, the RA volume fraction decreases, and the carbon content in the RA increases.

(3) Compared with Hollomon and modified C−J analysis, differential C−J analysis can better describe the work-hardening behavior of austempered bainitic steels. The work-hardening of tensile specimens exhibited three stages. The first stage mainly corresponds to the elastic deformation of RA, B, and M. In the second stage, the strain-induced phase transformation of RA to M mainly occurs. The third stage is the coordinated plastic deformation of all microstructures before tensile fracture. The difference in ductility is mainly reflected in the second stage, which depends on the initial content and distribution state of RA.

(4) Compared with austempered bainitic steels below Ms, the volume fraction of metastable RA in bainitic steels above Ms is higher, and metastable RA can effectively relieve local stress concentration by the TRIP effect during deformation, thereby increasing the total elongation of the austempered bainitic steel above Ms. This work will provide an experimental and theoretical basis for the development and application of high strength and toughness bainitic steel.

## Figures and Tables

**Table 1:** Chemical composition of the investigated steel (wt%).

C	Si	Mn	Ni	Cr	Al	Mo	Fe
0.30	1.52	0.26	1.51	0.27	1.10	0.25	Bal.

**Table 2:** Slope of different stages of three empirical models.

Sample	Hollomon Analysis, *n*		Differential C−J Analysis, (*n*_1_ − 1)			Modified C−J Analysis, (1 − *q*)	
	Slope 1	Slope 2	Slope 1	Slope 2	Slope 3	Slope 1	Slope 2
AT400	0.83	0.35	−0.19	−0.91	−21.01	−0.20	−3.99
AT350	0.95	0.20	−0.24	−4.00	−29.53	−0.25	−19.42
AT300	0.92	0.23	−0.22	−4.05	−30.56	−0.23	−18.14

## Data Availability

The raw/processed data required to reproduce these findings cannot be shared at this time as the data also forms part of an ongoing study.
